# Chili pepper preference development and its impact on dietary intake: A narrative review

**DOI:** 10.3389/fnut.2022.1039207

**Published:** 2022-12-14

**Authors:** Emily Siebert, Soo-Yeun Lee, Melissa Pflugh Prescott

**Affiliations:** ^1^Division of Nutritional Sciences, University of Illinois Urbana Champaign, Urbana, IL, United States; ^2^Department of Food Science and Human Nutrition, University of Illinois Urbana Champaign, Urbana, IL, United States

**Keywords:** capsaicin, chili pepper, appetite, spicy food, dietary intake, satiety, preference

## Abstract

A preference for chili pepper can be an acquired taste. The contrast between a chili lover and a hater illustrates the complexities involved in forming an appreciation for food that evokes a fiery pain sensation. This narrative review aims to understand the factors behind chili pepper preference formation across the life course and how individual chili pepper preferences can impact eating behaviors and dietary intake. This review was conducted using three databases, yielding 38 included articles. Results suggest five determinants of chili pepper preferences: culture, exposure, gender, genetics, and personality. Collective findings indicate that the strongest influences on preference acquisition include the individual environment from childhood to adulthood and repeated exposure to spicy flavors. With frequent exposure to spicy food, the perceived burn becomes less intense. Culture also influences exposure to chili peppers, with the highest consumption patterns seen within Mexico and some Asia countries. Additionally, males reported having a stronger preference for spicy foods than females. Twin studies illustrated that genetics influenced spicy taste preferences, underscoring the complexity of developing individual taste preferences. As for the impact of capsaicin-containing food on individual eating behaviors and dietary behaviors, appetite effects depend on the dose of capsaicin consumed, but three studies found a change in sensory desires for sweet and fatty foods after finishing a capsaicin-containing dish. Inconsistent results were reported for chili pepper's effects on hunger and satiety after consumption, but changes in specific food desires were observed. The impact of chili pepper on appetite and calories consumed was inconsistent, but the greater amount of capsaicin ingested, the greater the effect. Capsaicin's potential to be used for weight control needs to be further reviewed. In conclusion, evidence suggests that chili pepper preferences may be linked to innate and environmental aspects such as an individual's culture, gender, and genetics. Extrinsic factors like repeated exposure may increase the liking for spicy foods.

## Introduction

Chili pepper, a globally known spice that has been around for centuries, has no fat or calories but remains very flavorful. In 2018, the global pepper market was estimated at 4.1 billion dollars, with the highest consumption rates in Viet Nam, India, and the US (1). Spicy food trends are especially on the rise in the US, where an estimated 59% of consumers aged 18–34 prefer very spicy foods and demand a greater variety of spicy foods (2). Liking the spice from chili peppers is unusual compared to other foods because it elicits a pain response. This burning sensation comes from the compound capsaicin, which is detected by the body as a chemical irritant (3). Capsaicin activates the heat receptor TRPV1, a receptor on sensory nerve endings located not only in the mouth but across the whole body. The spicy sensation from capsaicin is not a flavor or taste but an irritant recognized within a pain pathway (4). TRPV1 is a part of thermoregulation, which allows humans to detect the burn of spicy foods, regulate core body temperature, and sense external temperature (5). Capsaicin is a part of a group of irritants that elicit a sensation of burning or tingling called chemesthesis. Chemesthesis is the detection of various chemicals by chemically sensitive pain and temperature receptors and is not recognized by the senses as taste or smell. This irritation by noxious chemicals stimulates the free nerve endings of the trigeminal nerve (CN V) in the oral cavity, the glossopharyngeal nerve (CN IX) in the back of the tongue, and the vagus nerve (CN X) in the airways and esophagus (6). Other examples of irritants similar to capsaicin include ginger, black pepper, wasabi, horseradish, or carbon dioxide from soda (7). Capsaicin may be an irritant but has been recognized as a potential anti-cancer agent and an anti-obesity compound (8). Its medicinal effects have also been linked to reduced satiety, and energy intake, (9) decreased abdominal fat and body weight (10), suggesting weight control benefits.

When consuming capsaicin from a pepper, the perceived hotness will depend on the type of pepper. The heat level of hot peppers is quantified using the Scoville scale in Scoville Heat Units (SHU) (11). Scoville Heat Units are determined based on a sugar water dilution process. The unit is in reference to how many times pepper had to be diluted for the burn to be undetectable. A bell pepper has a Scoville level of 0, a jalapeño pepper is ranked at around 4,000 units, and a ghost pepper is ranked at 1,041,000 units. The higher the units, the spicier the pepper. Since 1917, new methodologies have been used to quantify the pungency from capsaicin which is not as tedious as the sugar dilution process. For example, gas-liquid chromatography, gas chromatography, and gas chromatography-mass spectrometry are more common and reliable methods used today, but SHU is still used as a common reference point for the spiciness or pain from a pepper (12, 13).

Not only do we sense pain, but consuming capsaicin also involves temporal aspects. A spicy dish can be perceived as more or less spicy depending on the timing of the consumption. Sensitization occurs with rapid, repetitive consumption of spicy compounds within minutes and can cause the perceived burn of hot spices to seem spicier. Desensitization develops when there is a rest of at least 2.5 to 5 min and may last up to 24 h after the delayed ingestion of hot spice and can cause the perceived intensity of the spicy food to decrease (14, 15). Neurons become unresponsive in an extended refractory state of recovery from capsaicin and other spicy compounds (16). Therefore, having a spicy dish for lunch and having the leftovers for dinner can mean the same dish can be perceived as less spicy. These temporal aspects are important to consider when evaluating studies investigating spicy stimuli.

There are many theories behind spicy food preference acquisition that attempt to explain the variations in chili pepper or general spicy food popularity across regions and individuals. The thermoregulation theory proposes that the physiological response of sweating that occurs after eating a hot pepper helps consumers cool off in a hot climate (17). The antimicrobial theory postulates that general spices have been added to foods in hot climates to eliminate pathogens and provide health benefits (18). Bromham et al. disagree with this theory and claim that there is little evidence that spice consumption in hot countries reduces infection risk after evaluating the socioeconomic status, health-related statistics, and spice use in 93 countries (19). Another hypothesis regarding spicy preferences focuses on the personality of the consumers. The thrill-seeking theory suggests that those with adventurous personalities, driven by intense sensations or thrills, have a higher preference for spicy food or chili pepper (20). These theories, however, do not explain the extreme contrast between spicy food haters and spicy food likers, as well as the ability to grow a preference for the burn over time. Examples of extreme chili likers can be found in Mexico and many Asian countries. Some Mexican villagers claim that if they go too long without chili pepper, they crave it (21). In India, chili is the most consumed spice (22), and other Asian countries report 2.5–8 g daily chili pepper consumption per person (23), higher than American consumption, which is estimated to be at 1 g or less per person per day (23, 24). On the other hand, chili-dislikers in Japan and Europe do not feel that hot spices enhance the flavor of a dish, emphasizing the global contrast in spicy food preferences (25).

Genetics may also play a role in chili pepper preferences. The perceived intensity of the compound 6-n-propylthiouracil (PROP) has been used in research to provide insight into individual genetic sensitivity to bitter foods such as cruciferous vegetables, coffee, alcohol, or pungent spices such as capsaicin (26). The TAS2R38 gene has been shown to associate with PROP bitterness perception; however, capsaicin associates with PROP taster status but not TAS2R38 polymorphisms (27). Those who do not taste PROP are referred to as non-tasters and have fewer bitter taste receptors. In contrast, those who taste the bitterness of PROP are referred to as tasters (28). The extent to which these genetic variations influence taste perception for spicy foods, however, is not as clear. To address the gap in understanding how chili pepper preferences develop and the potential dietary consequences of spicy food intake, this narrative review explores two questions: (1) What factors influence chili pepper taste preferences during the life course? (2) How do preferences for chili pepper relate to overall dietary intake?

## Methods

In September 2021, an electronic literature search was conducted to gather more information on spicy taste preferences and the relationship between spicy food intake and dietary behaviors. Three databases were used, The Food Science Resource (FSTA) in Web of Science, Scopus, and PsycINFO. Search terms with Boolean functions captured in the title, abstract, or keywords included (prefer^*^ OR affinity OR lik^*^ OR accept^*^ OR desire^*^ OR accept^*^ OR enjoy^*^) AND (pregnan^*^ OR prenatal OR infan^*^ OR child^*^ OR adolescen^*^ OR Student$ OR Adult$ OR Human$ OR Subject$) AND (Spicy OR Pungen^*^ OR “hot spice^*^” OR Capsicum OR Capsaicin OR “spicy food” or “chili pepper” OR “hot pepper” OR “spicy flavor”) AND (develop^*^ OR cultur^*^ OR ethic^*^ OR tast^*^ OR consum^*^ OR frequen^*^ OR “dietary pattern” OR intake OR palatable OR palatability OR genetic$ OR gene$) AND NOT (cancer OR rat$ OR mice OR therap^*^). After removing duplicates, 963 abstracts and titles were screened by four undergraduate researchers. Inclusion criteria consisted of the following: articles must be based on original research, published in English, and must examine chili pepper consumption, intake, exposure, and preferences of hot spices of human research participants. Exclusion criteria included animal studies, food science and medicinal properties of peppers, or therapeutic properties of capsaicin for disease management and treatment.

## Results

Of the 38 articles that met the inclusion criteria, 28 provided insight into the first research question, eight provided insight into the second research question, and two answered both questions. Table 1 summarizes each article's findings related to the first research question. Table 2 summarizes each article's conclusions related to the second research question. Reviewed literature supported five common themes involved in chili pepper and spicy food preference formation across the life course: genetics, culture, exposure, gender, and personality. These individual factors will be further discussed in the following sections. In terms of the second research question and the relationship between chili pepper preferences and overall dietary intake, included literature explored the effects of chili pepper consumption on energy intake, self-reported cravings for other foods, and the impact on satiety and appetite.

**Table 1:** What factors influence chili pepper taste preferences during the life course?

**Author**	**Setting, *n***	**Objective**	**Experimental components**	**Key findings**
Rozin and Schiller (21)	US, Mexico, *n =* 395	To evaluate possible explanations that may account for the development of a liking for chili pepper	Chili pepper/capsaicin threshold, tolerance, and detection tests, interviews investigating chili pepper habits, preference tests for spicy snacks, and observations during mealtime in Mexican villager homes	American chili likers have a higher detection threshold for chili than dislikers (X = 8.04 for chili likers vs. X =7.44 for chili neutral/ dislikers). Slight, non-significant difference between Mexican (8.31 log_2_) and American (7.31 log_2_) thresholds. The correlation between tolerance and 5–16 years in age for Mexican subjects was *r* = 0.41, *p* < 0.05. Other age groups did not showcase significant threshold values
Guido et al. (29)	Italy, *n =* 203	To better define the relationships between both health and environmental factors on food preferences	5-point hedonic scale, demographic questionnaires	There is a trend for age and spicy food preferences; increasing age is associated with decreased spice preferences (.095 preference units, *P* < 0.001)
Rozin et al. (30)	US, *n =* 100	To determine the role of desensitization in preference acquisition	Threshold, salivary response, and sensitivity tests for capsaicin stimulus and chili liking questionnaires	Desensitization is a naturally occurring response following spicy food consumption but is not the leading cause of flavor preference formation. Those who have increased preferences for chili pepper have increased thresholds for it, *F* (2.44) = 5.00, *p* < 0.025
Rozin et al. (31)	US = 40	To determine if there is a relationship between liking and frequency, recent exposure and general chili pepper experience	Quantitative pleasure-ratings for varying spice degrees of cheese potato crackers, 5-point hedonic scale and chili-liking questionnaire	The hedonic report in the 1st min (when cracker flavor is still pleasant is enhanced by the presence of the burn). In slight-chili pepper likers, only weak burns produce this effect, and as liking increases, the level of burn that enhances the flavor increases
Lawless et al. (32)	US, *n =* 32	To determine if the capsaicin irritation would impact the perceived intensity of an olfactory stimulus and to determine the extent to which capsaicin irritation interferes with the flavor identification in groups with varying experiences with chili pepper/ capsaicin	Hot pepper liking and habit questionnaires, perceived intensity ratings, and identification task in a capsaicin or ethanol treatment trail	Chili likers perceive the burn of capsaicin as one-third less intense (x = 5.8) than non-eaters (x = 15.0). Negative correlation between burn intensity and consumption frequency scores (ρ = −0.60, *p* < 0.001) Composite chili pepper use and liking score (*p* = 0.52, *p* < 0.01). Perceived intensities for sucrose, NaCl, citric acid, and quinine decreased in capsaicin treatment trail for both chili pepper eaters and non-eaters F [1, 26] = 23.66
Logure and Smith (33)	US, *n =* 303	To identify if there are specific characteristics in humans that could predict individual food preferences	Questionnaires assessing food preferences, personality, and demographics	Males had a stronger preference for chili than females. M(SE) for women 4.9(0.2) and males 5.7(0.2), *p* ≤ 0.05. Correlation between age and chili food preferences (*r* = 0.14, *p* ≤ 0.05)
Rozin et al. (34)	US, *n =* 144	To verify the role of genetic factors in food preferences within monozygotic (MZ) and dizygotic (DZ) twins	Chili pepper, food attitude, and food preference questionnaires and comparison analysis within twin groups and random subject pairing	The only significantly detectable food preference difference between twin types was for preferred degree of hotness/ spicy in foods in monozygotic twins than dizygotic twins (MZ *r* = 0.44 DZ r = −0.08)
Nolden and Hayes (35)	US, *n =* 82	To generate a fixed dose-response curve function for varying concentrations of capsaicin to use in later spicy evaluation experiments while also differentiating how intensity ratings and liking for capsaicin may differ due to self-reported intake	Two separate visits evaluating self-reported perceived burn, bitterness, and liking/disliking ratings for four different capsaicin concentration levels (8 in total) in participants with varying levels of chili pepper intake	Self-reported estimation of chili pepper intake was significantly correlated with the reported liking of the burn (*r* = 0.37; *p* = 0.0005) and taste (*r* = 0.37; *p* =0.0005) of chili peppers suggesting those who consume chili peppers less report higher burn. Bitterness ratings were significantly associated with liking at the lowest capsaicin concentration, but bitterness was not correlated with liking for any other concentration (p's of other capsaicin concentrations >0.04)
Alley and Burroughs (20)	US, *n =* 148	To identify preference and aversion differences between males and females for unusual foods	Demographic and food questionnaires	Men (M = 2.66) had a stronger preference for hot and spicy condiments than women (M = 2.25), *t*(144) =3.15, *p* = 0.002. Men also reported greater use of hot peppers *t* (142) = 3.39, *p* < 0.001 and a preference for them *t* (139) = 4.67, *p* < 0.001 than women
Stevenson and Yeomans (36)	UK, *n =* 32	To investigate the relationship between chili pepper, burn intensity, and pleasantness in both those who like and dislike chili pepper to further understand liking development for chili pepper	Intensity and affective ratings for ascending concentration series of capsaicin using a visual analog scale	As time within the trial increases and the concentration of capsaicin increases, liking increases *F* (14.329) = 3.76. Females reported the burn as more intense than male subjects, with interaction sex x liking x trials being *F* (2.56) =3.48
Stevenson and Yeomans (37)	UK, *n =* 12	To identify the role exposure has in forming a preference for the burn of chili pepper	Experiment 1: Chili-level questionnaires for two groups consuming two different spice meals (2.5ppm vs 5.0ppm) once a week for five weeks. Experiment 2: 5 ppm meal given one week before the start of experiment 1 and one week after. Both utilized preference and hunger scales	Experiment 1: From week 1 to week 5, pleasantness ratings of both 2.5 and 5.0 spicy dish increased F (1, 10) =5.56, *p* < 0.05, Experiment 2: Liking and burn intensity rating correlation ρ = 0.41, therefore liking may not be due to reduced burn intensity
Törnwall et al. (38)	Finland, *n =* 331	To investigate the degree of influence that genetics and environment have on personal preference for spicy food/ oral pungency within monozygotic twins, dizygotic twins, and twins without their co-twin	Twin design study, sensory tests for rating pleasantness for capsaicin strawberry jelly, and spicy food preference questionnaire	Genetic influence explains 18–58% of liking for oral pungency, whereas 42–82% of the variation is attributed to environmental factors
Kim et al. (39)	Denmark, South Korea & US	To investigate the acceptance levels and hedonic reasoning for hot sauces and food pairings in consumers from different cultures	Test 1: Pizza and cream soup with fermented red pepper soybean pasted sauce and red pepper sauce compared to Tabasco sauce. Test 2: Grilled chicken and rice noodle soup with fermented red pepper soybean paste sauce, red pepper sauce compared to sriracha. Both tests assessed using hedonic and just-about-right (JAR) scale and check all that apply (CATA) method to evaluate internal drivers for liking/disliking samples.	48.5% of Danish consumers claimed to like the hot and spicy flavor of all sauces because it was new, 43.6% of American consumers liked it because it was familiar, while 50.9% liked the new flavor. 67% of Koreans claimed to like hot spice because it increased appetite, 37.7% of Koreans liked it because it relieved a stressful mood. As for sample specifics, all cultures agreed on ranking
Bègue et al. (34)	France, *n =* 114	To determine the relationship between spicy food eating behavior and level of salivary testosterone in all male subjects	Analysis of salivary testosterone of subjects self-serving spicy doses on their laboratory meal	Salivary testosterone was related to the number of selected spice doses (*r* = 0.294, *p* = 0.002) and evaluation of spiciness after consumption (*r* = 0.28, *p* = 0.003). Age was unrelated to the concentration of salivary testosterone (*r* = −0.11, *p* = 0.03), but related to the number of spicy doses selected (*r* = 0.19, *p* = 0.03) and general preference for spicy food (*r* = 0.18, *p* = 0.04)
Defrin et al. (40)	Israel, *n =* 115	To explore if temperature and pungency preferences are associated with either one's thermal sensitivity or ethnic origin-based pungency consumption	Warm and heat sensation measurements on tongue and hand. Self-reported preferred temperatures for eating, drinking, and bathing, and ethnic background and pungency preference questionnaires	The higher preferred degree of spiciness, the higher the preferred temperature for drinking (*r =* 0.44, *p* < 0.0001) and bathing temperature (*r =* 0.36, *p* < 0.0001). There were no significant differences between both parents born in countries with frequent consumption of spicy foods and those with both parents born with infrequent consumption (14/74 and 12/57%, *p* = 0.15)
Berry and Simons (41)	US, *n =* 59	To investigate the cultural differences in chili pepper consumption and preferences between Caucasian Americans and South Asian Indians	Chili pepper consumption frequency and spice preference surveys, with capsaicin stimuli irritation measures *via* general labeled magnitude scale (gLMS)	Many similarities were found between South Asian Indians and Americans for chili pepper liking and frequency, with almost identical chili liking scores (*P =* 0.88). No significant differences were noted for capsaicin irritation and capsaicin sensitivity between cultural groups (gLMS intensity *F*_1.55_ = 0.011, *P =*0.918)
Ludy and Mattes (42)	US, *n =* 25	To better understand the sensory, physiological, personality, and cultural differences between spicy food likers and non-likers to determine if there are any parallels between similar groups	Burn intensity assessed through subject evaluation of tomato soup with ascending concentrations of red pepper and psychophysical testing to assess oral, thermal, and auditory sensitivity, as well as personality and cultural attribute questionnaires	13 subjects classified as spicy food users who ate spicy food at least three times a week, ten of these users were men, and a higher portion of users reported earlier childhood spicy food consumption [*t* (11) = 3.074, *p =* 0.001]. Chili pepper users reported liking the taste and the burn more than non-users (all *p* < 0.001)
Zhang et al. (43)	China, *n =* 60	To investigate the relationships between age, sex, PROP ratings, consumption frequency of spicy foods, and pungent sensitivity to Sichuan pepper extract	Web-based questionnaires assessing demographics, health, spicy and salty food frequency, personality characteristics and PROP taster status	Significant age effects on pungency liking scores (*p* < 0.01). Younger and older age groups had significantly different thresholds for pungency (*p* < 0.05). The detection threshold of pungency was lower in females than male subjects (Females, 1.35e-03g/L) vs. (Males, 5.42e−03g/L)
Trachootham et al. (25)	Thailand & Japan, *n =* 168	To better understand how culture influences taste perception of both flavor and spice in Thai and Japanese subjects	Spicy food preference interviews using calibrated scoring methods comparing spiciness degree in culturally familiar curry along with recognition and detection thresholds for the five basic tastes	Japan had a higher percentage of participants with no spice preference compared to Thai subjects who had high spice preferences (*p* < 0.0001). A greater percentage of Japanese subjects consumed spicy food monthly, while a greater number of Thai subjects consumed spicy food weekly. Thai subjects had significantly higher taste thresholds on the anterior tongue and posterior tongue for the five tastes (sweet, salty, sour, bitter, and umami) than Japanese subjects (*p* < 0.0001)
Choi and Chan (44)	US, *n =* 350	To determine if there is a relationship between PROP taster status, hot chili pepper use, BMI, energy, and fat intake to assess the potential of spicy foods to promote weight loss	PROP taster status rated on a general magnitude scale, anthropometrics, chili pepper preference, and frequency questionnaires	Relationship between PROP intensity scores and chili pepper use was not significant (*P =* 0.322)
Castillo-Cardandang et al. (45)	Philippines, *n =* 3,072	To organize, describe and better understand food taste and cooking preferences in healthy adult Filipinos (20–50 years of age)	Community-administered questionnaires on taste and cooking preferences, demographics, and lifestyle behaviors	More males than females preferred spicy food (*p* < 0.001). Smokers preferred food to be spicy (*P =* 0.22), and more subjects < 40 years preferred spicy food than subjects aged 40–50 years (*p* < 0.0001)
Catanzaro et al. (46)	US, *n =* 139	To evaluate the relationship between food preferences and PROP tasting scores in college students	PROP ratings and enjoyment questionnaires for various foods, beverages, and different types of spicy foods (jalapeño peppers, crushed red pepper, spicy chili peppers) on a 5-point scale	Significant preference and PROP relationships for chili pepper *r =* −0.144, *p =* 0.46; spicy foods *r =* −0.083, *p =* 0.168; crushed red pepper *r =* −0.034, *p =* 0.348, and jalapeño peppers *r =* −0.049, *p =* 0.286
Spinelli et al. (47)	Italy, *n =* 1,146	To investigate how personality and taste responsiveness influence liking and choice of pungent foods and variability between males and females	Hedonic and perceived intensity ratings for different concentrations of tomato juice with capsaicin, PROP status, and questionnaires assessing chili liking, demographics, and personality: specifically, sensitivity to reward, punishment, disgust, private body consciousness, alexithymia, and food neophobia	Those more sensitive to reward had higher liking scores for the capsaicin sample. Those lower in food neophobia and disgust sensitivity liked the capsaicin samples more (*p* < 0.05)
Byrnes and Hayes (48)	US, *n =* 97	To determine the relationship between personality variables, factors, and the liking to different spicy food, response to the burn, and if sensation seeking is related to the frequency of chili consumption	The rated intensity of capsaicin stimuli and questionnaires related to food and chili liking and various personality measures: Private Body Consciousness, Sensation, Seeking, Sensitivity to Punishment, Sensitivity to Reward	Sensation seeking related to liking a spicy meal (*r =* 0.05, *p* < 0.0001) and frequency of chili consumption (*r =* 0.39, *p =* 0.0001) Perceived burn intensity was not related to personality measures
Byrnes and Hayes (49)	US, *n =* 246	To investigate the relationship between personality traits, perceived intensity capsaicin burn, and the liking and intake of spicy foods using the moderation model	Questionnaires related to food liking, chili liking generalized degree of liking and various personality measures: Arnett's Inventory of Sensation Seeking (AISS), Sensitivity to Punishment, and Sensitivity to Reward (SPSRQ)	Reported chili intake was positively associated with Sensation Seeking (*r =* 0.16, *p =* 0.02) and Sensitivity to Reward (*r =* 0.19, *p =* 0.005). However, the moderation model did not show that personality moderates the relationship between liking an intake expect for the trait Sensitivity to Punishment (β = 0.30, *p =* 0.02). Women showed a positive relationship with Sensitivity to Reward and reported yearly intake of chilies (*r* = 0.17, *p =* 0.04) and liking of a spicy meal (*r =* 0.21, *p =* 0.06) Men showed a moderate positive correlation with the liking of a spicy meal (*r =* 0.32, *p =* 0.004)
Byrnes and Hayes (50)	US, *n =* 103	To explore the relationship between risk-related behavioral personality measures and liking and intake of spicy foods and how these personality traits relate to one another	Hedonic rating of capsaicin-spiked strawberry jelly, self-reported race and ethnicity, questionnaires relating to Sensation Seeking (AISS) Sensitivity to Punishment, Sensitivity to Reward, degree of liking, chili pepper questionnaire, Balloon Analogue Risk Task	Sensation Seeking was significantly correlated with liking the μM 12 capsaicin sample was significant (*r =* 0.30, *p =* 0.002), liking the burn of a spicy meal (*r =* 0.24, *p =* 0.02). Risk-taking correlated with a yearly intake of spicy foods (*p =* 0.02).
Wang et al. (51)	China, *n =* 49,57,51	To examine the relationship between spicy taste and risk-seeking traits and behaviors	Study 1: Personality judgment task using the Chinese Facial Affective Picture System where neutral facial expressions were paired with a taste preference Study 2: Domain-Specific Risk-Taking Scale to assess inclination to task risks relating to gambling, health/ safety, recreational, social, and ethical Study 3: Iowa Gambling Task (IGT) to stimulate real-world decision-making under uncertainty and Positive and Negative Affect Schedule to report positive and negative feelings during a spicy and non-spicy condition	Study 1: Taste type affected judgments of irritability and risk-seeking Study 2: Liking spicy tastes positively correlated with a propensity to take risks (*r =* 0.37, *p* < 0.01) Study 3: Participants in the spicy group were more inclined to take risks during the IGT
Scott et al. (52)	US, *n =* 97	To evaluate how PROP taster status, personality factors, and emotions influence the perception and liking of tomato and butternut squash soups flavored with chipotle and ginger extracts	PROP ratings, intensity scales evaluating taste attributes and overall liking of three different concentrations of soup. Various questionnaires including the Universal Geneva Emotion and Odor Scale, general health, Arnett's Inventory of Sensation Seeking, and food preference questionnaire	Sensation seeking was associated with burn liking for chipotle chili tomato soup as the concentration of the chipotle chili extract increased (*F* (2.146) = 3.94, *p =* 0.02). PROP status had a non-significant effect (*p =* 0.10) of PROP taster status on liking ratings for chipotle chili tomato soup
Venkatramaiah and Devaki (53)	India, 38	To examine taste preferences as a function of certain personality traits in Indian culture	Indian Personality Inventory (IPI) which provides scores on the three guanas (traits) sattva (balance, joy, intelligence), rajas (energy, action, change), and tamas (darkness, inactivity, materiality) with a taste preference checklist on a 3-point scale to represent six traits salt (SLT), sweet (SWT), sour (SOR), bitter (BTR), bland (BLD) and (PNG)	Personality was associated with the six taste qualities *F* (2.10) = 4.32, *P* < 0.05. Tamas trait scored the highest for pungency (190/ 300) weighted score
Kwon (54)	Korea, *n =* 10,000	To evaluate the effects of estimated consumption levels of capsaicin from Korea's 2014–2018 NHANES dataset on its potential contribution to weight reduction and gastrointestinal distress	Estimated capsaicin levels based on 24-h recall interview data from NHANES database and capsaicin values from CAPKO database, and comparisons based on BMI, total energy intake, fat and sugar intake, age, and sex groups	Capsainoid consumption differed by males (3.94 ± 0.05) and females (2.94 ± 0.03) per day, but this difference decreased when accounting for body weight, with females reaching 79% of the consumption level of males compared to 63%

ρ, spearman r; n, sample size; M, mean; M(SE), mean squared error.

**Table 2:** How do preferences for chili pepper relate to overall dietary intake?

**Author**	**Setting, *n***	**Objective**	**Experimental components**	**Key findings**
Yoshioka et al. (55)	Canada, *n =* 23	To investigate the effects of red pepper/ capsaicin on feeding behavior	Study 1: Lunchtime macronutrient and energy intake assessment after red pepper high fat or high carb breakfast. Study 2: Energy and macronutrient intake assessments immediately after red pepper appetizer. Both utilized satiety and hunger scales	In study 1, red pepper did not affect the subjects' weight of ingested food or energy and macronutrient intakes at lunchtime. In study 2, the appetizer with red pepper decreased overall carb intake in lunch and mid-afternoon snacks by 18%, *P* < 0.05, and energy intake by 11%, *P* < 0.05
Reinbach, et al. (56)	Denmark, *n =* 40	To analyze how hot spices affect energy intake and appetite after subjects consumed a fixed portion meal with or without chili pepper every week for five weeks	Food intake (Kj), appetite, and liking were measured before, after, or sometimes during the meal on 9-point scales	Hot spices did not affect energy intake (*p* > 0.05) in the starter and buffet meals. After the chili-spiced meal, subjects had an increased desire to eat sweet foods (*p* = 0.041, adj *p* = 0.99)
Ludy and Mattes (42)	US, *n =* 25	To investigate the effects of commonly enjoyed red pepper doses in healthy young adults on appetite, energy expenditure, core body, and skin temperature	For three days before two testing visits, subjects followed a high fat (HF), high carbohydrate (HC) diet or their usual diet, keeping dietary intake records. Visits evaluated resting energy expenditure, core body and skin temperature, and appetite measurements during and after test meals with or without chili pepper	Desire to eat fatty foods was decreased more F(1.23) = 8.572, *p* = 0.008 in chili pepper nonusers than users in the 270 min after 1g red pepper test loads. Desire to eat salty foods decreased more *F* (1.23) = 9.922, *p* = 0.044 in chili pepper nonusers than users
Wen et al. (57)	China, *n =* 474,015	To explore the relationship between spicy food consumption and lifestyle behavioral characteristics in a large population size of adults from the 2004-2008 China Kadoorie Biobank Study	Surveys assessing lifestyle behaviors, eating, and drinking habits, demographics, spicy food consumption, and food frequency questionnaire	Among the reported regular spicy food consumers, the higher frequency reported, the stronger pungency preferred. Participants who preferred a stronger pungency degree were also more likely to consume meat, fresh vegetables, fresh fruits, soy products and had a higher energy intake, but were less likely to drink milk and soymilk (P for trend < 0.001)
Swint et al. (58)	US, *n =* 24	To compare how capsaicin and capsiate (non-spicy analog) from a meal would influence cravings, appetite, food intake, flavor liking, and physiological effects	Appetite, blood pressure, and energy intake measured at an ad lithium challenge meal (4.5 hours after capsaicin or no capsaicin test meal) self-reported dietary intakes recorded for the remaining day	Hedonic capsaicin-containing food questionnaire scores increased as the reported frequency of spicy food consumption increased (*p* ≤ 0.044) between all groups, and participants who consumed spicy foods at least monthly reported greater liking for the taste of chili pepper in food and were more likely to agree that chili pepper makes food taste better compared to those who consumed spicy foods less than once a month (*p* ≤ 0.021) between all groups
Andersen et al. (59)	Denmark, *n =* 66	To evaluate how tomato soup with cayenne pepper would influence the users' sensory-specific desires for sour, salty, sweet, bitter, spicy, fat, appetite/ hunger/ satiety ratings 4 hours post intake and overall psychological	Spicy or non-spicy soup compared against foods representing six sensory characteristics. Questionnaires of liking samples, hunger satiety, wanting for more portions, sensory satisfaction, and demographics	Differences in appetite and satiety were detected between two soups one-hour post intake, with the spicy soup resulting in lower ratings of hunger (*p* = 0.028) and higher ratings of satiety (*p* = 0.022). Adding cayenne pepper to tomato soup was found to
		wellbeing compared to a non-spiced soup		increase the desire to eat fatty and sweet foods (*p* < 0.001) and decrease the desire to eat salty and spicy foods (*p* < 0.0001)
Choi and Chan (44)	US, *n =* 350	To determine if there is a relationship between PROP taster status, hot chili pepper use, BMI, energy, and fat intake to assess the potential of spicy foods to promote weight loss	Anthropometrics, chili pepper preference, and frequency questionnaires to assess daily energy and fat intake	Chili pepper users had a higher calorie intake than chili pepper nonusers (*P* = 0.02), but fat intake did not significantly differ
Rigamonti et al. (60)	Italy, *n =* 10	To investigate how capsaicin capsules (2mg) administered orally after a meal effects energy intake and energy expenditure in young obese subjects in a singly blind randomized, placebo-controlled, crossover design	Anthropometrics, indirect calorimetry to obtain resting energy expenditure, blood samples to assess hunger and satiety hormones, and hunger and satiety evaluated by visual analog scale	PYY, GLP-1, ghrelin, insulin, and glucose did not differ between the capsaicin and placebo groups. Capsaicin significantly increased REE in the capsaicin but not placebo group (*P* < 0.05) comparing pre and post REE
Kwon (54)	Korea, *n =* 10,000	To evaluate the effects of estimated consumption levels of capsaicin from Korea's 2014-2018 NHANES dataset on its potential contribution to weight reduction and gastrointestinal distress	Estimated capsaicin levels based on 24-h recall interview data from NHANES database and capsaicin values from CAPKO database, and comparisons based on BMI, total energy intake, fat and sugar intake, age, and sex groups	Energy intake was significantly higher in the individuals categorized in the moderate high capsaicinoid intake (HC) and very high capsaicinoid intake (VHC) than in lower intake subgroups (*p* < 0.05). Fat intake was significantly higher in VHC groups compared with other subgroups in males in their 20's, 30's, 40's, and 50's and females in their 30s. Sugar intake was also significantly higher in the males 20's, 30's, 40's, 50's VHC groups and female VHC 20s and 40's compared with other subgroups (*p* < 0.05)

### Spicy food and culture

Six published articles evaluated how culture shapes food preferences or the role of home culture in spicy food or chili pepper consumption habits. This section includes findings related explicitly to spicy food preferences, individual sensitivity to capsaicin, and spicy food consumption within and across countries. In addition, the impact of various cultural food traditions on chili pepper consumption frequency, sensory perception of chili pepper, degree of preference, and initial exposures are discussed in detail below.

#### Chili pepper consumption frequency across cultures

Included literature evaluated consumption patterns for foods containing chili pepper in countries with varying cuisine types. In Rozin and Schiller's study, Mexican villager participants reported chili consumption at least three times a day, while American participants reported an average of 2.62 times per week (21). Comparatively, Berry and colleagues found that more than half of their Caucasian Americans (53.3%) subjects reported three to four times a week frequency, 40% reported daily consumption, and the remaining 6.7% reported consuming chili more than once a day (41).

As for Asian countries, 85% of Japanese adults reported monthly spicy food consumption compared to only 30% of older Thai adults. More Thai adults reported weekly spicy food consumption than the Japanese adult group (25). South Asian Indians consumed chili pepper more frequently within the month, with precisely 47.5% consuming chili pepper three to four times a week, 36% daily, and 16.5% more than once daily (41). Hot sauces are another way to make non-spicy food spicy and are most commonly consumed in Asia and the United States (39). Kim et al. (39) reported that 56% of the Korean subjects added hot sauce to 692 foods, while 91% of US participants applied hot sauce to 478 foods. Kwon reported the mean consumption of capsaicin based on Korea's NANES 2014–2018 data was 5.5 g/day, including both red pepper powder and fresh chili pepper, which is higher than the US and Europe (54). Choi and Chan's (44) evaluation of chili consumption patterns in an ethnically diverse American population found significantly more chili pepper users among Asian Americans compared to other ethnic groups. Ludy and Mattes (24) also recognized frequent spicy food use among Asian Americans. 53% of their participants classified as regular spicy food users were Asian Americans. But ethnicity, however, does not always predict spicy food use. One of Ludy and Mattes's (24) non-spicy food users was Asian American, and Defrin (40) found that subjects from different ethnic backgrounds in Israel did not differ significantly in their frequency of spicy food consumption, *p* = 0.15. These results suggest that spicy food consumption is high in some Asian countries, the US, and Mexico, but consumption frequency varies from person to person.

#### Ethnicity differences in thresholds/sensitivities

Sensory perception differences for capsaicin were also explored, specifically involving threshold sensitivity detection methods and perceived intensity analysis across different cultural groups for varying concentrations of capsaicin. Comparing Mexican and American differences, Rozin and Schiller (21) found a non-significant difference in chili pepper thresholds between the two ethnic groups, but Mexican thresholds were slightly higher (see Table 1). Berry and Simon's sensitivity test for capsaicin used a general magnitude scale with the following ratings: 0.78 being weak and 1.7 being very strong for a 100-ppm capsaicin solution. Caucasian Americans rated the average maximum intensity score 0.83 ± 0.05, while South African Indians rated the average maximum intensity score 0.82 ± 0.05, which were overall very similar (41).

#### Preference ratings by groups

The wide range of chili pepper preferences is evident within and across countries, especially within the Asian continent. Defrin et al. (40) conducted a study in Israel, and 54.5% of the subjects reported consuming spicy foods regularly, but 22.9% of the total sample preferred no spice, and the other 17.4% preferred the highest degree of spice. Contrasts for spicy preferences are seen between Thai and Japanese as well. Specifically, 70% of Thai subjects reported a mild to moderate spice preference, while 90% of Japanese subjects preferred no or mild spice (25). Differences are also evident within America but seem to be still influenced by ethnicity. In Ludy and Mattes (24) study, spicy food users agreed that chili pepper makes food taste better, as it tastes too bland without chili. Their reported spicy food users consisted of seven Caucasian Americans and six Asians. For Caucasian Americans in Berry and Simon's study, their chili pepper use and liking score calculated based on the sum of the number-coded responses for the online survey questionnaire was very similar to South Asian Indians, 34.6 vs. 34.7 on an 18 to 41 numerical scale (41). When comparing American preferences against Mexican villagers, Rozin and Schiller found that 68% of American subjects, aged 17–25, liked chili compared to 88% of Mexican subjects, aged 4–56 (21). Different degrees of spice preference are scattered across the globe and vary from region to region.

#### Starting age of consumption

When evaluating cross-cultural differences, a critical aspect to investigate is the consumer's initial spicy food exposure. When Rozin explored preference acquisition across Mexican and American cultures, all Mexican subjects reported that chili is introduced in small amounts at a young age, with the spice level increasing gradually. In contrast, first exposures to chili pepper varied across American subjects (21). A common theme across studies evaluating cross-cultural preferences and spicy sensitivities was starting age of consumption or childhood exposure. For US subjects, Swint et al. (58) found that the starting age of consumption for foods with chili pepper was 16.3 +/−3.2 for those who eat spicy food less than monthly, 9.9 +/−4.9 for those who eat spicy food monthly to weekly, and 10.4+/1 4.7 for those who consume spicy food three or more times weekly. In comparison, American chili likers in Rozin's (21) study reported more frequent use of hot spices by their parents. A trend for childhood consumption and preference for chili was also evident in Ludy and Mattes's study. They found that 69% of spicy food users reported consuming foods containing chili peppers from childhood [*t*(23) = 3.800, *p* = 0.001] (24). Another similarity for childhood exposure was also discussed in South Asian Indians, who are more likely to consume chili pepper since childhood than Americans, χ^2^ (2, *N* = 59) = 8.93, *P* = 0.003 (41). While Choi and Chan found that 80.2% of US spicy food consumers tended to be first exposed in early to late childhood (44).

### Exposure

The relationship between exposure and chili pepper preference has been a topic of interest by different scientists evaluating spicy preference development since 1981. Seven articles investigated how exposure to a spicy compound through multiple tastings or repeated exposure from the outside environment influences preference and eating habits. Stevenson and Yeomans (37) analyzed the role of repeated exposure in liking chili pepper burn. They found that the spicy meal was liked more in the fifth exposure than the first exposure but liking ratings between the first and the last fluctuated. Rozin and Schiller (21) explored initial exposure to chili pepper in their subjects, and subjects reported that 37% of their exposure came from the home or 29% of parents putting it in their food. Outside pressures to eat chili pepper were also mentioned, as 77% of Mexican mothers believed there was pressure from a friend or sibling to eat it (21). When looking at the chili liker vs. the chili non-liker, 46% of the American chili likers reported earlier exposure from their parents at home compared to only 6% of chili non-likers (21).

#### Exposure with age

A difference between chili likers and dislikers is the consumption of spicy food in childhood. Two studies recognized that those who started consuming chili pepper at a younger age reported using chili pepper more often in adulthood (24, 44). However, there were conflicting findings regarding aging and spicy food preferences. As depicted in Table 1, Guido and colleagues found an inverse relationship between age and spicy food preference, as younger subjects enjoyed spicy food more than elderly subjects (29). In contrast, Louge et al. (33) found that spicy food preferences had significant, positive correlations with age. However, Spinelli and colleagues reported that age was not associated with the frequency of chili consumption (47). The exact period when chili pepper preferences change was not discussed in Guido and Logue's studies. Rozin and Schiller (21), however, report that initially, chili pepper is aversive and is learned to be liked. There is a gradual increase in spicy preferences over the age range of 2–3 years to 8–9 years old in Mexican subjects (21). Mexican villager participants reported being exposed to gradually increasing amounts. Therefore, their preference change was seen as soon as age two or three (21). When evaluating pungency thresholds between younger and older age groups, younger age groups had a lower average detection threshold for pungent stimuli, 0.60e-03 g/L, whereas the older age group had an average detection threshold of 6.09e-03 g/L (43). Collective findings support that exposure to chili pepper in childhood promotes a preference for it.

#### Sensory characteristics and exposure

Seven studies reviewed how exposure to chili influences the level of burn perceived across different individuals and their tolerance to the burn. The more often chili pepper is consumed, the less intense the burn becomes (32, 35). This trend was confirmed in Stevenson and Yeoman's second experiment when their subjects reported lower burn ratings for the spicy stimuli in the last weeks of repeated exposure (37). Spinelli and colleagues also showed that chili non-users rated the burning intensity of capsaicin solution significantly higher than chili-users (47). Nolden (35) reports that frequency of use affects hedonic responses, with frequent chili users rating significantly higher concentrations of capsaicin than low-intake users (*p* < 0.05). In addition, it was found that spicy-food users could differentiate across the variety of burn intensities better than non-spicy users (24). However, the spicy-food users perceived the different capsaicin stimuli to be significantly spicier than the non-users, which is different than what Lawless et al. (31) detected. This difference in Ludy and Mattes (24) study could be due to more substantial desensitization effects in the non-users who are not as accustomed to tasting a burning food.

When strictly evaluating the liking of the burn from chili pepper, Rozin et al. (31) revealed that there is a liking for the burn, as hedonic reports are enhanced by the presence of the burn. As the liking for the burn increases, the level of burn that enhances flavor increases. With more exposure, the threshold for chili pepper slightly changes, with Rozin et al. (31) discovering that behavioral threshold was positively related to the frequency of consumption *r* = 0.39, *p* < 0.005. In addition to changes in threshold with more chili pepper consumption, salivary sensitivity decreased too, *r* = 0.49, *p* < 0.001 (30). A relatively weak relationship was also found between threshold and specific age ranges in the Mexican villagers. The correlation for chili threshold and age was *r* = 0.17 and *r* = 0.22 for ages 4–15 and 18–56, respectively (21).

#### Sensory characteristics between chili liker and disliker

The differences between a chili liker and a chili hater can provide valuable insight into how exposure changes sensory perception. Nolden and Hayes (35) found that their subjects who enjoyed spicy/very spicy food items reported a lower burn feeling than the other subjects sampling the same stimuli. Törnwall (38) also identified a similar trend, with non-likers perceiving pungent stimuli more intensely than the likers: *F*_2_._322_ = 5.3; *p* < 0.01. Stevenson and Yeomans (36) also discovered a similar pattern in their subjects who distinguished themselves as median chili likers to be able to sample up to a higher dose than other subjects who did not classify themselves as chili likers. This was also identified in Spinelli's study, where chili non-users rated capsaicin burning intensity significantly higher than chili users *p* < 0.0001 (47). However, one study contradicted this relationship. In Byrnes and Hayes' 2013 study, no correlation was observed between reported chili intake and burn intensity (*r* = 0.10, *p* = 0.89) and again in 2015, specifically between annual chili intake and perceived burn intensity, *r* = −0.05, *p* = 0.46 (49). Regarding threshold to spicy stimuli, Rozin et al. (30) also found differences between a solid spicy food liker and a neutral disliker. The mean threshold for the strong liker was 2.58 compared to 2.07, and the strong likers also had an increased salivation response (30). Chili likers can also tolerate higher concentrations of capsaicin, with the designated median chili liker sampling up to the 128-ppm dose compared to the median non-liker who only sampled up to the 16-ppm dose (36).

### Gender differences

Spicy food preference differences have been frequently evaluated between males and females. Eight articles discussed gender differences in spicy food intake, liking, and sensitivity. When comparing male and female chili pepper preferences, all but one study concluded that males have a stronger preference for spicy foods than females. On a 1 to 5 spicy food preference scale, Nolden and Hayes (35) found females to prefer a spice level of 1.8, while males preferred a level of 2.4, with 1 representing no heat preference and 5 representing very spicy. The overall spice levels (no heat/ mild, medium, spicy, very spicy) significantly differed by gender as well, CMH χ^2^ = 12.6; *p* = 0.01 (35). As shown in Table 1, Defrin, Logue, Smith, and Castillo-Carandang's findings also agreed with this trend, with males preferring a higher degree of spiciness compared with females (33, 40, 45). Alternatively, Stevenson and Yeomans (36) study found conflicting liking scores. Their results indicated that female chili likers had a higher mean visual analog scale (VAS) chili liking score than male chili likers, 93.7 compared to 85.9, on a scale of 0–100. The females and males categorized as non-likers both had a mean VAS chili-liking score of 36. When the two genders had to rate the increasing capsaicin doses, the female groups rated them more pleasant than the male groups. However, females rated the burn more intense than males (36). In contrast, Ludy and Mattes found no differences in perceived burn intensity ratings between males and females for the same spicy tomato soup (24). Yet, Zhang et al. (43) found different detection thresholds for capsaicin between the two genders. Females had a lower detection threshold at an average of 1.35e-03 g/L compared to males at an average of 5.42e-03 g/L. Differences in recognition thresholds for pungency were also evident, with females recognizing a dose as low as 6.73e-03 g/L on average and males detecting an average pungent quantity of 14.78e-03 g/L, *p* < 0.05 (43).

When analyzing consumption differences for spicy food between the genders, males generally consumed more spicy foods than females. For example, males reported a frequency of 71.4 times a year (+/−23.1) for hot sauces, whereas females reported 55.5 times per year (+/−11.1) on average. For red pepper flakes, males reported consuming this item 67.3 times a year (+/−15.2) on average, while females reported consuming 39.4 (+/−8.1) on average, but these differences written by Nolden and Hayes (35) did not reach statistical significance. In their study, Ludy and Mattes (24) also concluded that males more often eat chili peppers. Ludy and Mattes (24) divided subjects based on how frequently they use chili pepper, and the user group consisted of ten males and only three females. Scott et al. (52) also reported males consumed spicy food more frequently (*F* (1.74) = 8.10, *p* = 0.006), Kwon found that the average capsaicinoid consumption level was higher in males than females with female capsaicinoid consumption being 63% of that in males, but this value changes when accounting for body weight (Table 1) (54). Bègue and colleagues noticed this trend between males, females, and spicy food and concluded that testosterone could predict spicy food eating behavior. A positive correlation was found between the amount of salivary testosterone in male subjects and spontaneous spicy doses selected (Table 1) (34).

### Genetics and spicy food preferences

The genetic influence of liking spicy foods was discussed and reviewed in six research studies, including two twin studies. Törnwall, Rozin, and Millman conducted separate twin studies evaluating the influence of genetics on spicy/ pungency taste preferences. Both studies found similar patterns between monozygotic (MZ, identical) and dizygotic (DZ, fraternal) twins. Genetic effects for liking spicy food were minimal, but MZ twins did have stronger correlations for various pungency traits, such as pleasantness of spicy foods and spices, MZ *r* = 0.62, CI 95% (0.31, 0.79) DZ *r* = 0.25, (0.03, 0.44) (38). As shown in Table 1, Rozin and Milman's findings illustrate a similar trend, with MZ twins sharing a stronger correlation than DZ twins. Other traits were tested for correlation, too, including spicy food alone. MZ twins correlated *r* = 0.24, while DZ twins correlated *r* = −0.06 (61). Collective results suggest a slight genetic influence, but genetics are not the primary determinant of spicy food preferences.

PROP taste sensitivity has been proposed to affect chemesthetic perception (26). However, the literature in the present review has found weak relationships between PROP taster status and chili preferences. Törnwall et al. (38) found no significant correlations between PROP intensity scores and chili preferences/ users. Specifically, the Pearson r coefficient for PROP intensity and preference for mild pungency is −0.06, while the r coefficient for strong pungency preference is −0.01(38). Similarly, Choi and Chan also found no significant differences in PROP intensity scores between those who use chili pepper and those who do not. Still, they did see gender and ethnicity differences for PROP taster status. Female subjects were significantly more likely to be tasters than males. The Asian American subject group had the lowest number of non-tasters (18.5%) but the highest number of chili pepper users (44). In other words, the Asian American subject group reported the most chili pepper use. Still, the group consisted mostly of PROP tasters, meaning these individuals had more bitter receptors, but the majority of the group enjoyed spicy food. However, Spinelli reported that responsiveness to PROP was positively associated with burn intensity ratings, but chili user status did not affect PROP responsiveness (47). In Scott's study using spiced soups, non-tasters were found to like the soup with a high concentration of chipotle chili extract more than both medium-tasters and super tasters (*F* (2.74) = 3.96, *p* = 0.02), but this was not statistically significant (52).

Ludy and Mattes (24) also tested PROP taster sensitivity in spicy food users and non-users. They found that PROP scores did not relate to spicy food use; instead, it was exposure to spicy food in childhood that was the strongest predictor of spicy food consumption (24). However, Catanzaro and Chesbo did detect a significant relationship between spicy food and PROP taster status but noted that this was a low correlation of “little practical significance” (46).

### Personality traits and chili pepper liking

Eight articles explored the relationship between personality or behavior, chili liking, perceived burn, and intake frequency. Personality differences were detected between chili likers and non-likers. The traits investigated included sensitivity to disgust, food neophobia, sensitivity to punishment, sensitivity to reward, sensation seeking, body consciousness, variety, and risk-seeking. Spinelli found chili non-users were significantly more sensitive to disgust (*p* < 0.0001) more neophobic (*p* < 0.0001), more sensitive to punishment (*p* = 0.003), and less sensitive to reward (*p* = 0.003) than chili users (47). Byrnes and Hayes detected a similar trend, with sensitivity to reward positively correlating with liking a spicy meal (*r* = 0.23, *p* = 0.03) and a negative relationship between liking and sensitivity to punishment (*r* = −0.19, *p* = 0.06) (52). Chili non-users were also significantly more sensitive to disgust, more neophobic, more sensitive to punishment, and less sensitive to reward than chili users (47). Sensation Seeking was recognized in four studies as a personality trait associated with reported chili intake and chili liking; see Table 1 for correlation values (6, 48, 49, 52). Interestingly, high sensation seekers liked both the medium (*F* (1.74)=6.28, *p* = 0.01) and high (*F* (1.75) = 11.74, *p* = 0.001) concentrations more than the low sensation seekers (52).

In addition to chili liking, the frequency of chili consumption also had associations with personality traits. Reported frequency for combined chili intake was associated with individual variety-seeking scores [*F* (1.80) = 3.2; *p* = 0.07] (35). Males consumed spicy foods more frequently *F* (1.74) = 8.10, *p* = 0.006, and also had a high sensation seeking score (*F* (1.74) = 18.23, *p* < 0.0001) (52). Chili frequency intake had a weak positive relationship with sensitivity to reward and a moderate association with sensation seeking (52).

In terms of the perceived burn of chili pepper, all Byrnes and Hayes studies reported no correlation with any personality traits (6, 48, 49). However, Spinelli detected a weak but significant positive relationship between food neophobia and sensitivity to disgust and the perceived intensity of capsaicin solution (*p* < 0.05). However, Spinelli's sample size was much larger than Byrnes and Hayes' and consisted of Italians, compared to Americans. Overall, chili pepper users and likers correlated with the personality traits sensation seeking, sensitivity to reward, and propensity to take risks.

### Chili pepper effects on appetite and satiety

Six articles used various types of appetite scales to assess changes in appetite after exposure to different spicy stimuli. Three of these articles assessed appetite sensations using hunger and satiety scales. Swint et al. found no significant satiety differences following red pepper and capsaicin consumption compared to control meals or placebo capsules (55, 58, 60). However, Yoshioka et al. (55) study reported decreased appetite following red pepper ingestion. Andersen et al. (59) findings illustrated that participants who consumed the spicy soup reported higher satiation ratings and lower hunger ratings than participants who ingested the non-spicy soup immediately after intake (*p* = 0.002) and again 1-h post-meal. Specifically, satiation was reported as 6.13 (non-spiced soup) vs. 6.79 (spiced soup) on a nine-point scale, and hunger ratings were reported as 3.34 (non-spiced soup) vs. 2.93 for the (spiced soup) 1-h post intake also on a 9-point scale. In addition, the consumption of spicy soup was found to lower the desire to continue eating (59). Even though no significant effects on satisfaction were found in Swint's study, ratings for satisfaction were slightly higher for the spicy soup than the non-spicy soup, *p* = 0.002 (58). Ludy and Mattes (24) findings differed based on chili pepper use and experience. Ludy only saw hunger and appetitive effect differences in those who do not commonly eat red pepper. Specifically, hunger ratings decreased more (*F* (9.207) = 2.299, *p* = 0.018) in red pepper users after no red pepper. But the non-users who did consume red pepper reported reduced appetite characteristics, like preoccupation with food. Fullness ratings did not vary between red pepper and no red pepper stimulus (42). A possible reason appetite effects varied between studies could be due to the amount of capsaicin consumed in each study or the user's prior experience with spicy food. Ludy and Mattes (42) used one gram of red pepper, Rigamonti (60) used two mg capsaicin capsules, whereas Yoshioka et al. (55) used 10 g of red pepper in their test meal, and the subjects were all female. Reinbach et al. (56) used 0.6 g chili pepper and Swint et al. (58) used 1 g ground cayenne pepper. Additional research is recommended to support these findings.

### Chili pepper effects on food consumption

Chili pepper's effects on overall food consumption were assessed in various methods involving weight measurements for total food consumed, individual participant energy expenditure, and total energy intake during a meal or daily. Five studies analyzed the effects of spicy food on total food consumption. Three studies weighed the grams of food consumed at a meal, two calculated total calories consumed, two calculated energy expenditure, one estimated energy and fat intake using food frequency questionnaires, and one evaluated blood levels of orexigenic/anorexigenic peptides after a meal. All studies had conflicting results. Reinbach et al. (56) reported that adding hot spices to a meal slightly changed the total calories eaten and did not reach significance. Interestingly, men who consumed the spiced meal containing chili ate less than the non-spiced meal containing no chili, but women ate more of their spiced meal. However, these values did not reach statistical significance (56). In Yoshioka et al. (55) first study, adding red pepper to the experiment meals significantly decreased prospective food consumption immediately before lunch, *P* < 0.05. Specifically, subjects who ingested the meal with red pepper consumed 129–352 calories less based on the mean caloric values (55). Two other studies compared energy intake in individuals with varying chili eating habits and found similar trends depending on user status for chili pepper. Ludy and Mattes (42) observed that caloric intake tended to be lower (*F* (1.23) = 3.010, *p* = 0.096) in non-chili pepper users than in chili pepper users after both subjects consumed their preferred red pepper dose (mean decrease in calories = 43 kcals). Choi and Chan detected a similar trend, with chili pepper users consuming a significantly higher amount than non-users based on estimations from a food frequency questionnaire. Specifically, chili pepper users' mean energy intake was recorded to be 2,235.5 (55.3) kcals per day, *P* = 0.016, while chili pepper non-users mean energy intake was recorded to be 2,053.7 (51.0), *P* = 0.016 (44). In contrast, Swint and colleagues found no significant differences in food weight and calories consumed with or without red pepper, but this could be due to the low dose of hot spice chosen for the procedure. Ludy and Mattes explored a different route to evaluate red pepper's digestive effects and assessed the impact of 1 gram of red pepper on subjects' energy expenditure. Energy expenditure was higher (*F* (1.23) = 6.6944, *p* = 0.015) after consumption of red pepper compared to no red pepper, with a mean increase of 10 kcal over 270 min (42). In Rigamonti's (60) study, consuming 2 mg of a capsaicin capsule after ad lithium lunch significantly increased REE (from 1957.2 ± 455.1 kcal/day up to 2342.3 ± 561 kcal/d, *P* < 0.05; vs. placebo: from 2060.1 ± 483.4 kcal/d up to 2,296 ± 484.5 kcal/day), but postprandial levels of PYY, GLP-1, ghrelin, gastric, glucose, and insulin did not significantly differ between placebo and control (60). Although conflicting findings are present for capsaicin's effects on hunger and satiety, the two studies supporting an increase in energy expenditure suggest promising opportunities for capsaicin as an anti-obesity element.

### Spicy food influences cravings for other foods and macronutrient consumption differences

Many of these same studies also considered the effects of capsaicin on specific cravings, dietary habits, or overall macronutrient intake. Three studies analyzed how hot spices could impact specific sensory desires. Both Andersen et al. (59) and Reinbach et al. (56) observed that spicy stimuli increased subjects' desire to eat sweet foods. Reinbach et al. (59) statistical values for sweet cravings and spicy food consumption correlated *p* = 0.041 / adjusted *p* = 0.99 compared to Andersen's correlations *p* < 0.001. However, Ludy and Mattes (42) recognized a decreased desire to eat sweet foods in only non-chili pepper users after consumption of 1 g of red pepper (*F* (1.23) = 3.777, *p* = 0.064), as well as a decreased desire to eat fatty foods (*F* (1.23) = 8.572, *p* = 0.008), but just in non-chili-pepper-users. Andersen also recognized a decreased desire for fatty foods in all subjects after consuming spicy stimuli, *p* < 0.001 (59). Therefore, most studies agree that spicy foods can decrease cravings for fatty foods, except for one which noted a difference in cravings depending on chili-pepper user status. Yoshioka and colleagues also evaluated the effects of red pepper additions on macronutrient intake. They observed that red pepper in breakfast decreased protein intake by 20% in the high-fat meals and 6% in the high-carb meals at lunchtime, *p* < 0.05 in Japanese females (55). In contrast, Swint did not detect significant differences between test meals for fat, carbs, and protein for the rest of the study day after subjects consumed both capsaicin and capsiate at a test meal (58). These were the only two studies that analyzed macronutrient intake. One analyzed dietary patterns of almost half a million adults using surveys found that individuals who ate spicy food daily consumed the most poultry (102.1 grams/week) compared with other food categories. Additionally, the more often spicy food is consumed or, the stronger the preferred hot spice degree, the higher the proportion of snacking and increased preference for salty taste. Specifically, 22.8% of non-spicy food consumers compared to 38.9% of daily spice consumers (57).

## Discussion

The results of this literature review indicate that forming a preference for spicy food is linked to intrinsic and extrinsic factors. Gaining an appreciation for chili pepper could be due to a chronic desensitization effect, as frequent consumption leads to less perceived burn and decreased salivary sensitivity (30, 32). Although the degree of liking varies across cultures, gender, and age, acquiring an appreciation for the spicy flavor is possible with increased exposure (21, 31, 37, 58). It was also noted across three separate studies that those exposed early in their lifetime consume chili pepper more often in adulthood (39, 41, 42).

There were inconsistent results for capsaicin's effects on appetite behaviors and eating patterns. However, different doses of capsaicin were used across all studies, such as 10 g of red pepper (30 mg of capsaicin) (55), 1 g of red pepper (42), or as little as 0.6 g chili pepper (~0.375 mg of capsaicin) (56), which could attribute to the discrepancies in findings. In addition, participants varied in weight status and experience with spicy food which could also contribute to the differences in appetite responses to capsaicin illustrated in the literature. The body's regulation of feeding is a complex interplay between energy intake, body weight, and homeostasis control through gut hormones (62). Therefore, these individual differences could also account for the variations in appetite responses. A meta-analysis by Ludy et al. (63) suggests that capsaicin response depends on the dose consumed, and the capsaicin response inconsistencies could relate to differences in body composition as well. More specifically, it explained that differences in body composition might account for different thermogenic effects, such as appetite regulation and fat oxidation (63). This meta-analysis concluded that capsaicin and capsiate increase energy expenditure and enhance fat oxidation, especially at high doses. Still, the magnitude of these effects is small (63). These doses reached as high as 135 mg of capsaicin/ day/ participant, which related to changes in fat oxidation and resting energy expenditure but was linked to no changes in hunger or satiety (64). However, two studies in the present review noted appetite effect differences but at lower capsaicin doses. Specifically, Yoshioka's findings reported that consuming 10 grams of red pepper during breakfast decreased protein and fat consumption at lunchtime in females (55). Ludy's randomized crossover study found lower energy intake at lunch, but only in subjects who were not considered regular consumers of spicy food.

Swint et al. and Reinbach et al. (56, 58) found no effect on energy intake, food intake, or satiety ratings in the present literature review. Still, the amount of capsaicin used was much smaller compared to other studies, specifically 0.6 g of chili pepper (~0.375 mg) and 2 mg of capsaicin. While Ludy's meta-analysis finds that higher doses have the most substantial effects, it is not precisely understood how capsaicin can increase energy expenditure and influence fat oxidation. It is hypothesized that capsaicin's link to thermoregulation through the TRPV1 receptor can stimulate energy expenditure (65), and TRPV1 can control thermoregulation of the sympathetic nervous system (66). This SNS activation from capsaicin could favor fat oxidation (63). Capsaicin has recently been of interest to scientists as a potential substance to control appetite and weight loss (8). In this literature review, these studies only evaluate the effect of capsaicin short-term, such as the effects from one or two meals (55) or hours within one research visit (59). If capsaicin is considered for weight loss and appetite control, long-term effects should be further reviewed. However, spicy food consumption has been associated with decreased overall portion size as well as increased satisfaction following the meal (67) and is inversely associated with LDL cholesterol (68). Although, in contrast to this anti-obesity concept, Sun et al. (69) findings from the China Kadoorie study illustrate that eating spicy food had a positive association with adiposity measures in Chinese subjects, and daily spicy food eating was significantly associated with an increase of 0. 044 and 0.51 BMI (kg/m2) for both genders. The mechanisms of this correlation, however, were not addressed. Additionally, energy intake for chili pepper users was significantly higher than for non-chili users in Choi and Chan's study represented in this literature review. However, the authors suggest that PROP taste sensitivity contributes more energy intake than chili pepper use. These contradictory findings emphasize the need for additional research to be conducted (44). Choi and Chan also reported high chili pepper use in the Asian American participant subgroup, even though the majority of this group were PROP tasters. If PROP tasters are predicted to be more sensitive to capsaicin than the non-tasters, then the majority of spicy food consumers should be non-tasters. However, this was not the case here.

The mere exposure effect contends that people develop a preference for things that are more familiar to them (70). A review on the impact of cultural background on consumer perception and acceptability of foods and drinks explains that familiarity with a product can also predict liking (71). Therefore, it is expected that those exposed to chili pepper initially in childhood or within the home are more inclined to consume it in adulthood, which was observed in three studies in this literature review (24, 41, 44). Culture also influences what we are exposed to in our childhood. Reddy et al. demonstrate in their review discussing the cultural influence of nutrition, that cultural habits are often developed in childhood and are difficult to change since the individual internalizes them (72). Collectively, this suggests that childhood exposure to chili pepper can predict spicy food use and preferences later in life. Additionally, consuming chili pepper daily has been proposed to cause a long-term desensitization effect, as the nerve endings consistently become desensitized (30). In Stevenson and Prescott's study, consumption of a spicy solution for 2 weeks decreased perceived burn intensity in participants. Capsaicin is not a taste but a nerve response, so it is not unusual for the nerves to desensitize or fatigue when frequently responding to spicy stimuli (73, 74). Interestingly, capsaicin may also modulate the texture or taste perception of food, such as the sweetness from sucrose (75) or the saltiness of NaCl (76). Lyu et al. (77) found that capsaicin affects thickness discrimination and perception of soups, regardless of the subject's habitual capsaicin use, yet perceived burn intensity differed between chili users significantly at the 1 and 10 ppm concentrations. This difference between chili users agrees with the findings in this literature review. Spicy food preferences have also been reported as a factor affecting weight loss in head and neck cancer patients after radiation. Patients with a strong spicy preference experienced a higher percent weight loss after radiation than those with a milder preference. Strong spicy food lovers had the lowest energy intake and highest requirement of tube feeding after radiation (78). This relationship could provide insight into how spicy food impacts the oral mucosa and the importance of screening patients for spicy taste preferences before radiation.

These collective findings suggest that spicy food can be less painful with more consumption and more enjoyed once repeatedly exposed. Repeated exposure is often used as a strategy for kids to eat foods they initially do not like, which is similar to the pattern of spicy food. Spicy food originally is aversive to some due to the pain. Still, as noted in this literature review, it can be learned to be liked and influence mealtime food consumption and sensory desires.

Our cultural experiences also impact how we view and eat food (79), which aligns well with the findings of this literature review. Similar consumption patterns were recognized within cultural groups, but individual taste preference differences are inevitable. Twin studies have shown that genetics partly influence food choice and dietary intake (51). Two twin studies in the present review described correlations between genetics and spicy taste preferences, but no genetic relationship was established between PROP taster status and spicy food preference in the three studies presented here. Outside this literature review, Ullrich et al. found that PROP tasters who are traditionally predicted to dislike strong-tasting foods liked hot sauce and chili peppers when identified as food adventurous on a personality test (28). Within this review, personality has been shown to correlate with spicy food preference, suggesting that personality traits such as sensation seeking, propensity to take risks, and sensitivity to reward may be a stronger predictor of spice preference than PROP taster status (48–50, 52, 80).

### Limitations and strengths

This literature review highlights the factors involved in spicy preference development and how these preferences can impact eating behavior, but limitations must be considered. Eleven out of the 38 studies were published before 2010, and only five were published in the past 3 years. Although it is essential to consider the evolution of spicy food preference acquisition findings, scientific understanding has evolved since 1980, and new scientific techniques have been discovered (81). Additionally, spicy snacks are more common and widely available across America, so eating habits recorded before 2010 most likely do not reflect common eating habits today. Most of the studies evaluating the effects of capsaicin on food intake were randomized control trials, which are not always generalizable to real-life settings and behaviors. It is critical to acknowledge that eating behaviors are driven by personal, social, cultural, environmental, health, and economic factors (82). Publication bias is another element that should be considered since this literature review only includes published original research. These results may not reflect all the experiments relating to this topic, and published results may favor positive results. One key strength of this review was that it included studies conducted on all continents except Australia and Antarctica. When evaluating spicy taste preferences across the life course, it's critical to scrutinize a global perspective, and 15 different countries were included in this review.

## Conclusions and implications for practice, policy, and research

Collective findings illustrate the many factors contributing to spicy taste preference development. We found that many ethnicities consume chili pepper in their dishes, and those who consume chili pepper more often have a stronger liking than those who do not. Repeated exposure can increase liking, and initial exposure in childhood favors chili pepper use in adulthood. Personality traits such as sensation seeking and willingness to take risks are also associated with an appreciation for chili pepper. These findings illustrate the diversity of individuals who enjoy spicy food despite its pain and emphasize the influence of both nature and nurture in shaping food preferences. Understanding the reasons behind liking spicy food may be necessary for the food industry and food service demands. Results for the effects on dietary intake, however, were less clear. Chili pepper in meals has been shown to change sensory desires for salty, sweet, and fatty foods, but energy intake, appetite, and satiety results were mixed; however, two studies supported capsaicin's increasing effect on energy expenditure. Discerning how spicy food impacts dietary intake can also provide insight into the food choice and health outcomes relating to spicy food use. Chili pepper remains an anti-obesity component of interest, but future studies are needed to clarify how commonly consumed capsaicin doses impact appetite and satiety and the potential mechanisms involved.

## Author contributions

ES presented the research questions to MP and S-YL and wrote the manuscript with support from S-YL and MP. MP provided supervision. MP and S-YL provided edits. All authors contributed to the article and approved the submitted version.

## References

[1] World - Pepper - Market Analysis Forecast Size Trends and Insights. Available online at: https://www.researchandmarkets.com/reports/4701016/world-pepper-market-analysis-forecast-size (accessed August 19, 2022).

[2] Trend Insight: Sweet Heat. FONA. Available online at: http://www.fona.com/articles/2020/10/trend-insight-sweet-heat (accessed August 24, 2022).

[3] Cho H Kwon Y. Development of a database of capsaicinoid contents in foods commonly consumed in Korea. Food Sci Nutr. (2020) 8:4611–24. 10.1002/fsn3.1785 32884741 PMC7455983

[4] Szolcsányi J. Effect of capsaicin on thermoregulation: an update with new aspects. Temp Multidiscip Biomed J. (2015) 2:277–96. 10.1080/23328940.2015.1048928 27227029 PMC4843897

[5] Montell C Caterina MJ. Thermoregulation: channels that are cool to the core. Curr Biol. (2007) 17:R885–7. 10.1016/j.cub.2007.08.016 17956748

[6] Dalton P Byrnes N. Psychology of chemesthesis - why would anyone want to be in pain? In: Chemesthesis: Chemical Touch in Food and Eating. Wiley. (2016). p. 8–31. Available online at: https://www.scopus.com/inward/record.uri?eid=2-s2.0-85041585559&doi=10.1002%2f9781118951620.ch2&partnerID=40&md5=e72652c9e236a4d5a48412df05869423

[7] Lawless HT. Chapter 8 - Flavor. In: Friedman MP, Carterette EC, editors. Cognitive Ecology. San Diego: Academic Press (1996) p. 325–80. (Handbook of Perception and Cognition (Second Edition)). Available online at: https://www.sciencedirect.com/science/article/pii/B9780121619664500104 (accessed October, 22 2022).

[8] Zheng J Zheng S Feng Q Zhang Q Xiao X. Dietary capsaicin and its anti-obesity potency: from mechanism to clinical implications. Biosci Rep. (2017) 37:BSR20170286. 10.1042/BSR20170286 28424369 PMC5426284

[9] Van Avesaat M Troost FJ Westerterp-Plantenga MS Helyes Z Le Roux CW Dekker J. Capsaicin-induced satiety is associated with gastrointestinal distress but not with the release of satiety hormones. Am J Clin Nutr. (2016) 103:305–13. 10.3945/ajcn.115.123414 26718419

[10] Snitker S Fujishima Y Shen H Ott S Pi-Sunyer X Furuhata Y. Effects of novel capsinoid treatment on fatness and energy metabolism in humans: possible pharmacogenetic implications. Am J Clin Nutr. (2009) 89:45–50. 10.3945/ajcn.2008.26561 19056576 PMC3151435

[11] Scoville WL. Note on capsicums. J Am Pharm Assoc. (1912). 1:453–4. 10.1002/jps.3080010520

[12] Bensinger PH Biftu T. Determination of pungency due to capsicum by gas-liquid chromatography. J Food Sci. (1977) 42:660–5. 10.1111/j.1365-2621.1977.tb12573.x

[13] Usman MG Rafii MY Ismail MR Malek MdA Latif MA. Capsaicin and dihydrocapsaicin determination in chili pepper genotypes using ultra-fast liquid chromatography. Molecules. (2014) 19:6474–88. 10.3390/molecules19056474 24853712 PMC6271280

[14] Green BG. Capsaicin sensitization and desensitization on the tongue produced by brief exposures to a low concentration. Neurosci Lett. (1989) 107:173–8. 10.1016/0304-3940(89)90812-4 2616028

[15] Green BG. Temporal characteristics of capsaicin sensitization and desensitization on the tongue. Physiol Behav. (1991) 49:501–5. 10.1016/0031-9384(91)90271-O 1648242

[16] Fischer MJM Ciotu CI Szallasi A. The mysteries of capsaicin-sensitive afferents. Front Physiol. (2020) 11:4195. 10.3389/fphys.2020.554195 33391007 PMC7772409

[17] Lee TS. Physiological gustatory sweating in a warm climate. J Physiol. (1954) 124:528–42. 10.1113/jphysiol.1954.sp005126 13175196 PMC1366289

[18] Billing J Sherman PW. Antimicrobial functions of spices: why some like it hot. Q Rev Biol. (1998) 73:3–49. 10.1086/420058 9586227

[19] Bromham L Skeels A Schneemann H Dinnage R Hua X. There is little evidence that spicy food in hot countries is an adaptation to reducing infection risk. Nat Hum Behav. (2021) 5:878–91. 10.1038/s41562-020-01039-8 33542529

[20] Alley TR Burroughs WJ. Do men have stronger preferences for hot, unusual, and unfamiliar foods? J Gen Psychol. (1991) 118:201–14. 10.1080/00221309.1991.9917781 28142506

[21] Rozin P Schiller D. The nature and acquisition of a preference for chili pepper by humans. Motiv Emot. (1980) 4:77–101. 10.1007/BF00995932

[22] Siruguri V Bhat RV. Assessing intake of spices by pattern of spice use, frequency of consumption and portion size of spices consumed from routinely prepared dishes in southern India. Nutr J. (2015) 11:7. 10.1186/1475-2891-14-7 25577292 PMC4326183

[23] Gonlachanvit S. Are rice and spicy diet good for functional gastrointestinal disorders? J Neurogastroenterol Motil. (2010) 16:131–8. 10.5056/jnm.2010.16.2.131 20535343 PMC2879848

[24] Ludy MJ Mattes RD. Comparison of sensory, physiological, personality, and cultural attributes in regular spicy food users and non-users. Appetite. (2012) 58:19–27. 10.1016/j.appet.2011.09.018 21986186

[25] Trachootham D Satoh-Kuriwada S. Lam-ubol A Promkam C Chotechuang N Sasano T. Differences in taste perception and spicy preference: a thai-japanese cross-cultural study. Chem Senses. (2018) 43:65–74. 10.1093/chemse/bjx071 29136162

[26] Duffy VB Bartoshuk LM. Food acceptance and genetic variation in taste. J Am Diet Assoc. (2000) 100:647–55. 10.1016/S0002-8223(00)00191-7 10863567

[27] Nolden AA McGeary JE Hayes JE. Differential bitterness in capsaicin, piperine, and ethanol associates with polymorphisms in multiple bitter taste receptor genes. Physiol Behav. (2016) 156:117–27. 10.1016/j.physbeh.2016.01.017 26785164 PMC4898060

[28] Ullrich NV Touger-Decker R O'sullivan-Maillet J Tepper BJ. PROP taster status and self-perceived food adventurousness influence food preferences. J Am Diet Assoc. (2004) 104:543–9. 10.1016/j.jada.2004.01.011 15054337

[29] GuidoDPernaSCarraiMBaraleRGrassiMRondanelliM. Multidimensional evaluation of endogenous and health factors affecting food preferences, taste and smell perception. J Nutr Health Aging. (2016) 20:971–81. 10.1007/s12603-016-0703-427925136

[30] Rozin P Mark M Schiller D. The role of desensitization to capsaicin in chili pepper ingestion and preference. Chem Senses. (1981) 6:23–31. 10.1093/chemse/6.1.23

[31] Rozin P Ebert L Schull J. Some like it hot: A temporal analysis of hedonic responses to chili pepper. Appetite. (1982) 3:13–22. 10.1016/S0195-6663(82)80033-0 7103463

[32] Lawless H Rozin P Shenker J. Effects of oral capsaicin on gustatory, olfactory and irritant sensations and flavor identification in humans who regularly or rarely consume chili pepper. Chem Senses. (1985) 10:579–89. 10.1093/chemse/10.4.579

[33] Logue AW Smith ME. Predictors of food preferences in adult humans. Appetite. (1986) 7:109–25. 10.1016/S0195-6663(86)80012-5 3740828

[34] Bègue L Bricout V Boudesseul J Shankland R Duke AA. Some like it hot: testosterone predicts laboratory eating behavior of spicy food. Physiol Behav. (2015) 139:375–7. 10.1016/j.physbeh.2014.11.061 25462592

[35] Nolden AA Hayes JE. Perceptual and affective responses to sampled capsaicin differ by reported intake. Food Qual Prefer. (2017) 55:26–34. 10.1016/j.foodqual.2016.08.003 28392628 PMC5383096

[36] Stevenson RJ Yeomans MR. Differences in ratings of intensity and pleasantness for the capsaicin burn between chili likers and non-likers implications for liking development. Chem Senses. (1993) 18:471–82. 10.1093/chemse/18.5.471

[37] Stevenson RJ Yeomans MR. Does exposure enhance liking for the chilli burn? Appetite. (1995) 24:107–20. 10.1016/S0195-6663(95)99328-2 7611746

[38] Törnwall O Silventoinen K Kaprio J Tuorila H. Why do some like it hot? Genetic and environmental contributions to the pleasantness of oral pungency. Physiol Behav. (2012) 107:381–9. 10.1016/j.physbeh.2012.09.010 23010089

[39] Kim HJ Chung SJ Kim KO Nielsen B Ishii R O'Mahony M. cross-cultural study of acceptability and food pairing for hot sauces. Appetite. (2018) 123:306–16. 10.1016/j.appet.2018.01.006 29325771

[40] Defrin R Dekel-Steinkeller M Urca G. Some like it hot: Preference for temperature and pungency consumption is associated with sensitivity to noxious heat. Eur J Pain. (2021 F) 25:473–84. 10.1002/ejp.1686 33089561

[41] Berry DN Simons CT. Do Caucasian American and South Asian Indian cultural groups differ in sensitivity to capsaicin? A study designed to control for chili pepper affinity. J Food Sci. (2020) 85:2896–901. 10.1111/1750-3841.15369 32794204

[42] Ludy MJ Mattes RD. The effects of hedonically acceptable red pepper doses on thermogenesis and appetite. Physiol Behav. (2011) 102:251–8. 10.1016/j.physbeh.2010.11.018 21093467 PMC3022968

[43] Zhang LL Zhao L Zhang QB Shi BL Zhong K Wang HY. The effect of the pungent sensation elicited by Sichuan pepper oleoresin on the sensory perception of saltiness throughout younger and older age groups. Food Qual Prefer. (2020) 86:3987. 10.1016/j.foodqual.2020.103987

[44] Choi SE Chan J. Relationship of 6-n-propylthiouracil taste intensity and chili pepper use with body mass index, energy intake, and fat intake within an ethnically diverse population. J Acad Nutr Diet. (2015) 115:389–96. 10.1016/j.jand.2014.09.001 25441957

[45] Castillo-Carandang NT Sison OT Velandria FV Sy RG Llanes EJB Reganit PFM. You are what you eat:” Self-reported preferences for food taste and cooking methods of adult filipinos (20-50 years old). Acta Med Philipp. (2014) 48:56–61.

[46] Catanzaro D Chesbro EC Velkey AJ. Relationship between food preferences and PROP taster status of college students. Appetite. (2013) 68:124–31. 10.1016/j.appet.2013.04.025 23648895

[47] Spinelli S De Toffoli A Dinnella C Laureati M Pagliarini E Bendini A. Personality traits and gender influence liking and choice of food pungency. Food Qual Prefer. (2018) 66:113–26. 10.1016/j.foodqual.2018.01.014

[48] Byrnes NK Hayes JE. Personality factors predict spicy food liking and intake. Food Qual Prefer. (2013) 28:213–21. 10.1016/j.foodqual.2012.09.008 23538555 PMC3607321

[49] Byrnes NK. The Influence of Personality and Experience on the Perception, Liking, and Intake of Spicy Foods. Dissertation Abstracts International: Section B: The Sciences and Engineering (2015).

[50] Byrnes NK Hayes JE. Behavioral measures of risk tasking, sensation seeking and sensitivity to reward may reflect different motivations for spicy food liking and consumption. Appetite. (2016) 103:411–22. 10.1016/j.appet.2016.04.037 27137410 PMC4902730

[51] Hasselbalch AL Heitmann BL Kyvik KO Sørensen TIA. Studies of twins indicate that genetics influence dietary intake. J Nutr. (2008) 138:2406–12. 10.3945/jn.108.087668 19022965

[52] Scott NO Burgess B Tepper BJ. Perception and liking of soups flavored with chipotle chili and ginger extracts: efects of PROP taster status, personality traits and emotions. Food Qual Prefer. (2019) 73:192–201. 10.1016/j.foodqual.2018.11.009

[53] Venkatramaiah SR Devaki PB. Personality traits and taste preference: An empirical study. J Pers Clin Stud. (1990) 6:1–5.

[54] Kwon Y. Estimation of dietary capsaicinoid exposure in Korea and assessment of its health effects. Nutrients. (2021) 13:2461. 10.3390/nu13072461. Available online at: https://www.scopus.com/inward/record.uri?eid=2-s2.0-85110333497&doi=10.3390%2fnu13072461&partnerID=40&md5=44cc1a04fb8abc4e9e8263e42fb9c9df 34371974 PMC8308769

[55] Yoshioka M St-Pierre S Drapeau V Dionne I Doucet E Suzuki M. Effects of red pepper on appetite and energy intake. Br J Nutr. (1999) 82:115–23. 10.1017/S0007114599001269 10743483

[56] Reinbach HC Martinussen T. Effects of hot spices on energy intake, appetite and sensory specific desires in humans. Food Qual Pref . (2010) 21:65561. 10.1016/j.foodqual.2010.04.003

[57] Wen Q Wei Y Du H Lv J Guo Y Bian Z. Characteristics of spicy food consumption and its relation to lifestyle behaviours: results from 05 million adults. Int J Food Sci Nutr. (2021) 72:569–76. 10.1080/09637486.2020.1849038 33207985 PMC8404681

[58] Swint JM Beining KM Bryant JA Tucker RM Ludy MJ. Comparison of capsaicin and capsiate's effects at a meal. Chemosens Percept. (2015) 8:174–82. 10.1007/s12078-015-9188-5

[59] Andersen BV Byrne DV Bredie WLP Møller P. Cayenne pepper in a meal: Effect of oral heat on feelings of appetite, sensory specific desires and well-being. Food Qual Prefer. (2017) 60:1–8. 10.1016/j.foodqual.2017.03.007

[60] Rigamonti AE Casnici C Marelli O De Col A Tamini S Lucchetti E. Acute administration of capsaicin increases resting energy expenditure in young obese subjects without affecting energy intake, appetite, and circulating levels of orexigenic/anorexigenic peptides. Nutr Res. (2018) 52:71–9. 10.1016/j.nutres.2018.02.002 29530622

[61] Rozin P Millman L. Family environment, not heredity, accounts for family resemblances in food preferences and attitudes: a twin study. Appetite. (1987) 8:125–34. 10.1016/S0195-6663(87)80005-3 3592649

[62] Wilding JPH. Neuropeptides and appetite control. Diabet Med J Br Diabet Assoc. (2002 A) 19:619–27. 10.1046/j.1464-5491.2002.00790.x 12147141

[63] Ludy MJ Moore GE Mattes RD. The Effects of capsaicin and capsiate on energy balance: critical review and meta-analyses of studies in humans. Chem Senses. (2012) 37:103–21. 10.1093/chemse/bjr100 22038945 PMC3257466

[64] Lejeune MPGM Kovacs EMR Westerterp-Plantenga MS. Effect of capsaicin on substrate oxidation and weight maintenance after modest body-weight loss in human subjects. Br J Nutr. (2003) 90:651–9. 10.1079/BJN2003938 13129472

[65] Janssens PLHR Hursel R Martens EAP Westerterp-Plantenga MS. Acute effects of capsaicin on energy expenditure and fat oxidation in negative energy balance. PLoS ONE. (2013) 8:e67786. 10.1371/journal.pone.0067786 23844093 PMC3699483

[66] Alawi KM Aubdool AA Liang L Wilde E Vepa A Psefteli MP. The sympathetic nervous system is controlled by transient receptor potential vanilloid 1 in the regulation of body temperature. FASEB J. (2015) 29:4285–98. 10.1096/fj.15-272526 26136480 PMC4650996

[67] Menghini M Singh R Thyagarajan B. Understanding food preferences and their connection to health perception among lean and non-lean populations in a rural state. Innov Pharm. (2020) 11:3449. 10.24926/iip.v11i4.3449 34007657 PMC8127119

[68] Xue Y He T Yu K Zhao A Zheng W Zhang Y. Association between spicy food consumption and lipid profiles in adults: a nationwide population-based study. Br J Nutr. (2017) 118:144–53. 10.1017/S000711451700157X 28673367

[69] Sun D Lv J Chen W Li S Guo Y Bian Z. Spicy food consumption is associated with adiposity measures among half a million Chinese people: the China Kadoorie Biobank study. BMC Public Health. (2014) 14:1293. 10.1186/1471-2458-14-1293 25518843 PMC4320519

[70] Hekkert P Thurgood C Whitfield TWA. The mere exposure effect for consumer products as a consequence of existing familiarity and controlled exposure. Acta Psychol. (2013) 144:411–7. 10.1016/j.actpsy.2013.07.015 24012724

[71] Jeong S Lee J. Effects of cultural background on consumer perception and acceptability of foods and drinks: a review of latest cross-cultural studies. Curr Opin Food Sci. (2021) 42:248–56. 10.1016/j.cofs.2021.07.004

[72] Culture and its influence on nutrition and oral health. Biomed Pharmacol J. (2015) 8:613–20. 10.13005/bpj/757

[73] Su T Gao X Li H Zhang L Han P Chen H. Frequent spicy food consumption is associated with reduced capsaicin and salty taste sensitivity but unchanged sour taste or intranasal trigeminal sensitivity. Food Qual Prefer. (2022) 96:104411. 10.1016/j.foodqual.2021.104411

[74] Prescott J Stevenson RJ. Pungency in food perception and preference. Food Rev Int. (2009) 11:665–98. 10.1080/87559129509541064

[75] Prescott J Stevenson RJ. Effects of oral chemical irritation on tastes and flavors in frequent and infrequent users of chili. Physiol Behav. (1995) 58:1117–27. 10.1016/0031-9384(95)02052-7 8623010

[76] Gilmore MM Green BG. Sensory irritation and taste produced by NaCl and citric acid: effects of capsaicin desensitization. Chem Senses. (1993) 18:257–72. 10.1093/chemse/18.3.257

[77] Lyu C Schijvens D Hayes JE Stieger M. Capsaicin burn increases thickness discrimination thresholds independently of chronic chili intake. Food Res Int. (2021) 149:110702. 10.1016/j.foodres.2021.110702 34600694

[78] Vittayakasemsont K Klaitong C Phukosi K Chavasit V Sinthusek T Trachootham D. Association between pretreatment dietary preference and weight loss after radiation therapy in head and neck cancer patients: a pilot study. Nutr Cancer. (2019) 71:230–9. 10.1080/01635581.2019.1578393 30836019

[79] Sato W Rymarczyk K Minemoto K Wojciechowski J Hyniewska S. Cultural moderation of unconscious hedonic responses to food. Nutrients. (2019) 11:2832. 10.3390/nu11112832 31752310 PMC6893624

[80] Wang X Geng L Qin J Yao S. The Potential Relationship Between Spicy Taste Risk Seeking, Judgment Decision Making. Sociated for Judgement Decision Making European Associaion for Decision Making. Vol. 11. (2016) p. 547–53. Available online at: https://journal.sjdm.org/vol11.3.html

[81] Fattori V Hohmann MSN Rossaneis AC Pinho-Ribeiro FA Verri WA. Capsaicin: current understanding of its mechanisms and therapy of pain and other pre-clinical and clinical uses. Molecules. (2016) 21:844. 10.3390/molecules21070844 27367653 PMC6273101

[82] LaCaille L. Eating Behavior. In:Gellman MD Turner JR, editors. Encyclopedia of Behavioral Medicine. New York, NY: Springer (2013). p. 641–2. 10.1007/978-1-4419-1005-9_1613

